# 

**Published:** 1872-04

**Authors:** 


					﻿Vol. VIII.
A.T>JEtIL, 1872.
No. 1.
THE BISTOURY.
JL QUARTEliT.Y JOURNAL,
Devoted to the Exposition of Charlatanism in Medicine and the Educa-
tion of the People upon Medical Subjects !
Thad S. Up De Graff, M. D„ Editor.
Terms of Subscription, Fifty Cents per annum, in Advance.
ELMIRA, N. Y.
PRINTED FOB THE PBOPBIETOB, AT THE DAILY ADVEBTISEB JOB PRINTING HOUSE.
				

